# Delayed life-threatening subdural hematoma after minor head injury in a patient with severe coagulopathy: a case report

**DOI:** 10.4076/1757-1626-2-7587

**Published:** 2009-08-10

**Authors:** Marc Engelen, Paul J Nederkoorn, Marion Smits, Diederik van de Beek

**Affiliations:** 1 Department of Neurology, Academic Medical Center, University of Amsterdam1100 DD, AmsterdamThe Netherlands; 2 Department of Radiology, Erasmus MC - University Medical Center RotterdamRotterdamThe Netherlands

## Abstract

Minor head injury is a frequent cause for neurologic consultation and imaging. Most patients with minor head injury will make an uneventful recovery, but in a very small proportion of these patients life threatening intracranial complications occur. We describe a patient on oral anticoagulation therapy, and severely impaired coagulation, with normal head computed tomography on admission, who developed a subdural hematoma requiring surgery 12 hours later. Current guidelines and literature for the management of minor head injury are discussed.

## Introduction

Minor head injury is a frequent cause for neurologic consultation in the emergency room. Most patients with minor head injury will make an uneventful recovery, but in a very small proportion of these patients life threatening intracranial complications requiring neurosurgical intervention do occur [1-3]. An interesting subgroup are patients with minor head injury who are on anticoagulant therapy. The NICE guidelines state it is most likely safe to discharge these patients if imaging is normal [4].

A guideline from the Dutch Neurology Association recommends clinical observation for at least 24 hours, even with normal findings on initial head CT [5]. In our experience, many Dutch clinicians feel that this is probably too strict and tend to deviate from this recommendation. Here we describe a patient on anticoagulant therapy who developed a subdural hematoma several hours after admission for minor head injury.

## Case presentation

**Figure 1.**
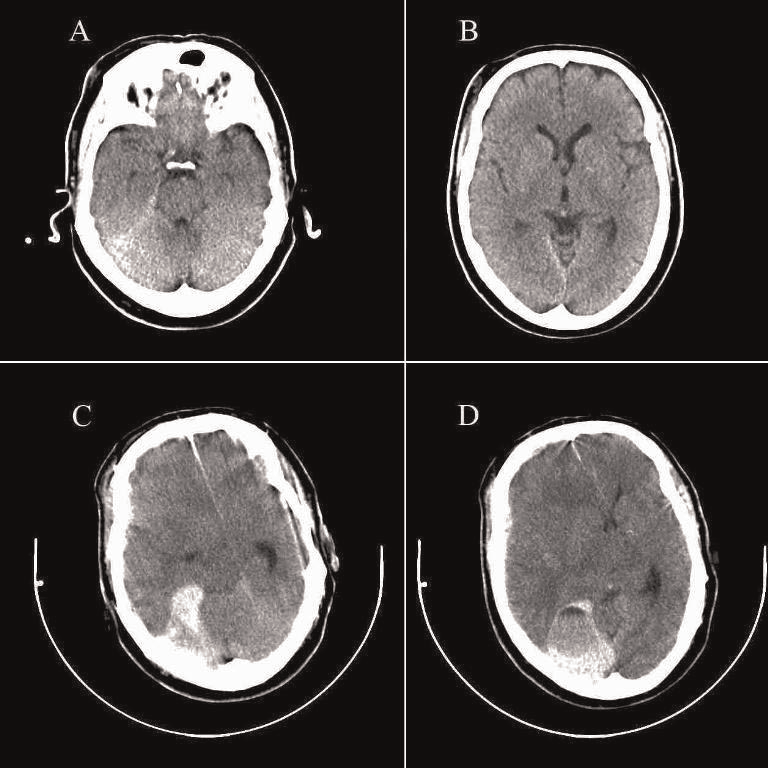
Non contrast enhanced CT scan at initial presentation on the emergency room **(A and B)** and 12 hours after admission **(C and D)**.

A 57 year old Dutch Caucasian man was beaten by a group of youngsters. He had suffered brief loss of consciousness and was brought to the emergency room. He had a history of a mechanical aortic valve replacement and was therefore using acenocoumarol. He had no neurologic complaints and neurologic examination was unremarkable with a Glasgow Coma Scale score of 15. The laboratory workup revealed an INR of 5.1, much higher than the target of 2.5- 3.5. Initial head CT on the Emergency Room was read as normal (Figure 1A and B). He was admitted to the neurological medium care unit for clinical observation according to our current guidelines [4,5]. Neurologic examination was normal 10 hours after admission, but 12 hours after admission he suddenly complained of severe headache and subsequent developed a rapidly decreasing level of consciousness. On neurologic examination he now had a score of 9 on the Glasgow Coma Scale and a dilated right-sided pupil that was unresponsive to light. Emergent head CT scan showed subdural hematoma around the tentorium cerebelli compressing the brain stem and causing substantial midline shift (Figure 1C and D). Coagulation was normalized with 2500 IU of human protrombin complex and 10 mg vitamin K, and subsequently the patient underwent neurosurgery for evacuation of the hematoma. He made an excellent recovery.

## Discussion

Our case illustrates the devastating late complication in a patient with minor head injury on anticoagulation therapy, with an INR above the target level of anticoagulation, and an initial normal head CT. In our patient, clinical observation allowed rapid evacuation of the subdural hematoma.

The idea that an early CT after minor head injury may be false negative for neurosurgical lesions is well recognized, and there are numerous reports on these so-called delayed hematomas. However, the incidence has been shown to be extremely low (<0.02%) [6], indicating that the sensitivity of CT for detection of neurosurgical lesions approaches 100% and CT may therefore be used safely to triage minor head injury patients for clinical observation. In a recent study of discharge after initial normal head CT versus clinical observation after mild head injury, no differences in outcome between these groups were found on follow-up [7]. Also, there were no complications requiring neurosurgical intervention in the group discharged after normal initial head CT in this study [7].

In our patient, the preliminary reading of the CT scan was without abnormalities, while the second scan, performed after late clinical deterioration, showed an infratentorial subdural hematoma. The detection of infratentorial subdural hematoma on CT is difficult due to partial volume averaging effects and scanning artifacts related to the osseous skull. Small hyperdense streak lesions, indicating a small subdural hematoma, on the initial CT scan may thus have falsely been interpreted as the commonly encountered beam hardening artefacts. Such difficulties are present in any patient, and it is likely that small infratentorial subdural hematomas commonly remain unrecognized. In our patient, however, coagulation was - iatrogenically - severely impaired, and the small subdural hematoma that would have been clinically inconsequential under normal circumstances, turned into a life-threatening complication.

Oral anticoagulation is a well-recognized risk factor for intracranial traumatic complications after minor head injury. In a Dutch multicenter study of 3181 patients with minor head injury, oral anticoagulation was found to have an odds ratio of 2.4 for abnormal CT [8], but did not seem to alter outcome if the initial head CT is normal [9]. In this study, 243 (7.6%) patients had intracranial traumatic CT findings and 17 (0.5%) underwent neurosurgical intervention [3]. This study has led to the so-called CT in Head Injury Patients (CHIP) prediction rule, consisting of a set of risk factors including anticoagulant therapy, to select patients for head CT [8]. Nowadays, head CT is readily available in the emergency setting and the question arises whether subsequent admission to a neurological ward is necessary. Prediction rules such as the CHIP rule, as well as the New Orleans Criteria [1], and the Canadian CT Head Rule [2], can be used in clinical practice to triage patients for CT and subsequent discharge if CT and neurological examination are normal [7].

From the presented case, the question arises whether such policy is justified for all patients with minor head injury, particularly in those with severely impaired coagulation. Previous studies have not specifically addressed this issue. The number of patients on anticoagulants that was included in the Dutch study was small (218 of 3181 [7%]). The level of anticoagulation, however, was not taken into account, as blood coagulation tests were not formally recorded in this study. Delayed intracranial complications after mild head injury can be devastating and fatal if they occur outside the hospital. Guidelines to manage minor head injury will need to find a reasonable balance between use of health care resources, and detecting rare but serious complications. Our case report underlines the need for a more individualized approach towards patients with risk factors that increase their risk of complications severely, even if initial assessment is normal.

## Abbreviations

CT: computed tomography
INR: international normalized ratio
